# Haematology nurses' perspectives of their patients' places of care and death: A UK qualitative interview study

**DOI:** 10.1016/j.ejon.2019.02.003

**Published:** 2019-04

**Authors:** Dorothy McCaughan, Eve Roman, Alexandra G. Smith, Anne C. Garry, Miriam J. Johnson, Russell D. Patmore, Martin R. Howard, Debra A. Howell

**Affiliations:** a Epidemiology & Cancer Statistics Group, University of York, York, YO10 5DD, UK; b Department of Palliative Care, York Hospital, York, YO31 8HE, UK; c Wolfson Palliative Care Research Centre, University of Hull, Hull, HU6 7RX, UK; d Queen's Centre for Oncology and Haematology, Castle Hill Hospital, Hull, HU16 5JQ, UK; e Department of Haematology, York Hospital, York, YO31 8HE, UK

**Keywords:** Haematological malignancy, Haematology nurse, Place of care, Place of death

## Abstract

**Purpose:**

Patients with haematological malignancies are more likely to die in hospital, and less likely to access palliative care than people with other cancers, though the reasons for this are not well understood. The purpose of our study was to explore haematology nurses' perspectives of their patients’ places of care and death.

**Method:**

Qualitative description, based on thematic content analysis. Eight haematology nurses working in secondary and tertiary hospital settings were purposively selected and interviewed. Transcriptions were coded and analysed for themes using a mainly inductive, cross-comparative approach.

**Results:**

Five inter-related factors were identified as contributing to the likelihood of patients’ receiving end of life care/dying in hospital: the complex nature of haematological diseases and their treatment; close clinician-patient bonds; delays to end of life discussions; lack of integration between haematology and palliative care services; and barriers to death at home.

**Conclusions:**

Hospital death is often determined by the characteristics of the cancer and type of treatment. Prognostication is complex across subtypes and hospital death perceived as unavoidable, and sometimes the preferred option. Earlier, frank conversations that focus on realistic outcomes, closer integration of palliative care and haematology services, better communication across the secondary/primary care interface, and an increase in out-of-hours nursing support could improve end of life care and facilitate death at home or in hospice, when preferred.

## Highlights

• Patients with haematological malignancies are more likely to die in hospital than people with other cancers.
• No previous research has reported on UK haematology nurses' perspectives of their patients' place of care and place of death.
• Hospital deaths were largely attributed to disease characteristics, nature of treatment and difficulties with prognostication.
• However, other modifiable factors were also identified as barriers to death at home.

## 1 Introduction

There are over sixty haematological malignancy subtypes (Arber et al., 2016; Swerdlow et al., 2016), and these ‘blood cancers’ are often categorised as leukaemias, lymphomas and myeloma. While the different diseases vary in presentation, prognosis and management, they share a common origin, arising from abnormal blood and bone marrow cells (Howard and Hamilton, 2013). Collectively, haematological malignancies account for around 10% of all new cancers diagnosed in England (Office for National Statistics, 2016). Although many are indolent (e.g. follicular lymphoma and chronic lymphocytic leukaemia), and behave like chronic diseases, others (e.g. acute myeloid leukaemia) are often aggressive (NICE, 2003).

Management varies by subtype: some are curable with intensive, toxic chemotherapy, associated with long periods of hospitalisation; others are incurable from diagnosis and managed with intermittent or continuous oral chemotherapy (NICE, 2003). Clinical pathways and prognosis depend on the disease, its response to treatment, and the patient's characteristics (Roman et al., 2016; Smith et al., 2015, 2018a). Illness trajectories may include lengthy periods of remission and relapse, as well as the potential for sudden deterioration, and the transition towards the last weeks or days of illness can be unclear (Button et al., 2017; Smith et al., 2010). Prognostication is notoriously difficult (Auret et al., 2003; Hui et al., 2016; Odejide et al., 2014), which has significant implications for the timely organisation and delivery of end of life care (LeBlanc and El-Jawahri, 2015).

Patients with haematological malignancies are more likely to die in hospital than people with other cancers, both in the UK and elsewhere (Dasch et al., 2016; Howell et al., 2010; National Cancer Intelligence Network, 2011); they are also less likely to be referred to specialist palliative care and hospice services (Adsersen et al., 2017; Earle et al., 2008; Howell et al., 2011; Hui et al., 2012), and when they are, it is often close to the time of death (Hui et al., 2014; LeBlanc et al., 2015). A key concern is that patients with blood cancers may end life amid aggressive, intensive treatment, still hoping for cure, and without palliative care input (LeBlanc, 2018; LeBlanc et al., 2017). Specialist palliative care be absent despite a high symptom burden resulting from bone marrow failure and psychological distress (Hochman et al., 2018; Manitta et al., 2011; Zimmermann et al., 2013).

Delivery of quality care to patients at the end of life, and understanding where best that care should take place, have long been prioritised on national and international research agendas (Higginson, 2016; Morris et al., 2015; WHO, 2004). Recent studies are beginning to question the desirability and feasibility of home death (Fjose et al., 2018; Hoare et al., 2015; Milligan et al., 2016) and challenge assumptions regarding delivery of palliative care in acute hospitals (Robinson et al., 2014, 2015, 2016). Results from a large-scale Australian study (Eagar et al., 2018), for example, show that symptom outcomes were better for patients receiving palliative care in hospital than at home, although the reasons for these differences were unclear.

The views of haemato-oncologists and palliative care specialists concerning end of life care for blood cancer patients are increasingly reported (Hui et al., 2015; McCaughan et al., 2018; Odejide et al., 2014; Wright and Forbes, 2014), yet little is known about the perspectives of haematology nurses, including their perceptions of reasons for the predominance of hospital deaths. The dearth of European research in this area, identified a decade ago by Grundy and Ghazi (2009), still prevails. Elsewhere, we identified only two publications, based on qualitative interviews with Australian nurses (McGrath and Holewa, 2006, 2007), which revealed concerns about the intensive nature of hospital-based treatments at the end of life, and lack of integration with palliative care. However, these findings were published over a decade ago, and their applicability to current UK practice is unclear.

The views of specialist nurses are crucial, however, due to their key role in discussing end of life care with patients and families and facilitating preferences (Lobban and Perkins, 2013). Our study therefore aimed to elicit the views of UK haematology nurses on their patients’ places of care and death. These interviews comprise one strand of a larger qualitative study, designed to capture the views of doctors and nurses working in haematology and specialist palliative care settings (McCaughan et al., 2017, 2018), as well as relatives of deceased patients (McCaughan et al., 2019). The study findings are reported in a series of cross-referenced publications, in order to accommodate the broad range of perspectives emanating from the different groups of participants.

## 2 Methods

### 2.1 Study design

Methods are described below using consolidated criteria for reporting qualitative studies (COREQ) (Tong et al., 2007). A qualitative descriptive approach (Sandelowski, 2000) was adopted to gain new understanding and insights into a phenomenon about which little is known (Pope and Mays, 2006). Semi-structured interviews were selected as they facilitate flexibility in data collection (Creswell, 2007), and generate ‘rich’ narratives (Flick, 2014). Methods were consistent with the interpretivist paradigm; we sought to understand the subjectivity of nurses' perspectives within the complexity of social processes (Broom and Willis, 2007).

### 2.2 Setting and participant recruitment

The study was conducted within the UK's Haematology Malignancy Research Network (HMRN: www.hmrn.org) (Smith et al., 2018b). This is a unique collaboration between university academics, National Health Service (NHS) clinicians, patients and carers, that facilitates research using a range of methods, including qualitative studies, with the purpose of generating evidence to improve clinical practice. As we sought interviewees who could provide rich data, we purposively selected (Patton, 2015) experienced haematology nurses from within the HMRN area, based on their role, years of practice and years of haematology nursing. Thirteen nurses were informed about the study by email, sent an information sheet, and asked to contact the researcher if they wanted to take part. Eight were interviewed, across four secondary and tertiary care hospitals; five agreed but could not take part due to work commitments. Interviewees were either ward/area managers or clinical nurse specialists, with between nine and 31 years nursing, and seven and 18 years haematology experience; some had transplant experience and others were skilled in working with patients from particular diagnostic groups. All had provided care for patients dying from their disease, and their families and friends, and had organised end of life care in different settings.

### 2.3 Data collection

Interviews were carried out during 2014, by DH, in private, at the nurses' places of work. A semi-structured topic guide (Table 1) was developed from existing literature and experiences of the study team; it was used flexibly to allow for unanticipated responses. Issues explored evolved as the study proceeded, with early insights informing subsequent interviews (Ritchie and Lewis, 2003). Data collection ceased after eight interviews that captured a wide range of nurses’ perspectives relating to the research questions, drawn from their varied experiences of working in different clinical settings (Baker and Edwards, 2012). Interviews lasted 45–90 min and, with participant consent, were audio-taped and transcribed verbatim.

**Table 1:** Interview topic guide.

Haematology nurses were asked about their perspectives on:
• place of care and death in their patients • reasons why hospital deaths predominate • factors preventing and promoting care and death at home • factors associated with the primary/secondary care interface • factors affecting whether people achieve their preferred place of care and death • issues specific to haematology compared to other conditions • changes that could facilitate death at home or closer to home

### 2.4 Analysis

Analysis involved qualitative description (Sandelowski, 2000), based on thematic content analysis, an approach considered well-suited to questions relevant to practitioners and policy makers (Green and Thorogood, 2018, page 258). Two researchers undertook data analysis (DM, DH), both qualified nurses, experienced in qualitative methods in applied health services research and haematology. Interviews were summarised through dynamic engagement with the dataset, while staying close to participants' accounts (Sandelowski, 2000). Analysis was mainly inductive: we drew on the data to generate meaning, with the aim of transforming the ‘raw’ data into a new and coherent depiction of the phenomena under scrutiny (Thorne, 2000). Guided by the research questions, our approach to data analysis balanced both inductive and deductive orientations (Green and Thorogood, 2018, page 252).

Transcripts were read and re-read for familiarisation, and initial codes (units of meaning) and themes identified. These were then expanded and modified through a reflexive and interactive process of ‘interrogating’ the data, in the search for common patterns and deviant/negative cases (Pope et al., 2000). Electronic spreadsheets were used to facilitate data summary and to enable easy comparison within and between cases. We aimed to move towards a detailed, nuanced interpretive account of the findings (Sandelowski, 2010), through analytic processes that facilitated data synthesis and interpretation. Reflective notes and memos formed the basis of regular discussions (DM, DH) to agree and refine codes and emerging themes. Discussing disagreements helped to refine data coding and interpretation, thereby enhancing rigour in the analytic process (Barbour, 2001).

## 3 Ethical approval

NHS ethical approval was obtained (Yorkshire and The Humber REC: 11/YH/0306). Participants were given verbal and written information about the study aims and their involvement and were told they could withdraw at any time. Written consent was sought from individuals prior to interview and assurances given concerning confidentiality and anonymity.

## 4 Findings

**Fig. 1:**
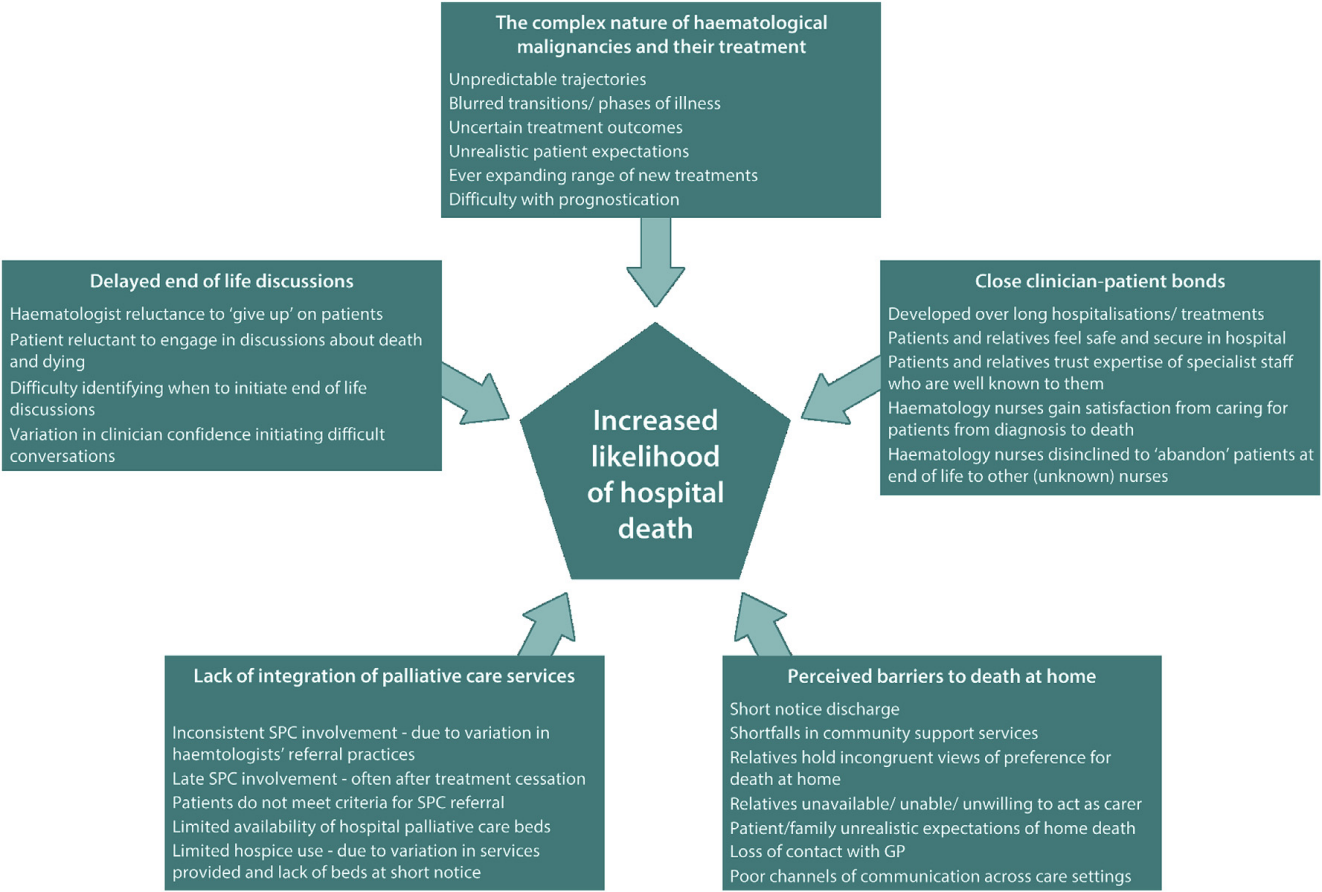
Summary of interrelated factors perceived as influencing likelihood of hospital death in patients with haematological malignancies.

The haematology nurses perceived hospital death as likely to predominate amongst their patients due to a complex interplay of interrelated factors. Five themes were identified that explicate these factors: (i) the complex nature of haematological malignancies and their treatment; (ii) close clinician-patient bonds; (iii) delayed end of life discussions; (iv) lack of integration of palliative care services; and (v) barriers to death at home. Inter-related factors perceived as influencing likelihood of hospital death are summarised in Fig. 1 and described below, with verbatim quotes.

### 4.1 The complex nature of haematological malignancies and their treatment

Hospital death was often attributed to the complex characteristics and unpredictable pathways associated with haematological malignancies. Nurses noted that the possibility of cure at a late stage, as well as the potential for sudden deterioration and death at any point in the trajectory, made it difficult for haematologists to estimate prognosis and so identify the ‘right’ time to stop or de-escalate treatment, and initiate end of life discussion and planning. Patients with aggressive disease, who often experience prolonged and intensive treatment in hospital, were considered particularly prone to rapid death. However, patients with indolent pathways were also considered susceptible to sudden decline due to the onset of sepsis or bleeding, which can act as a ‘trigger’ for hospital (re-)admission and result in hospital death.

*‘I had somebody who was palliative and they collapsed at home, there was a 999 and they ended up through A&E in ICU – they didn't want to come into hospital, they didn't want IV antibiotics, they didn't want escalated treatment and wanted to die at home’* (Haematology Nurse 4)

Several nurses compared the trajectories of haematological and non-haematological cancers, commenting that the latter tended to be more predictable, with linear progression towards terminal disease, and clearer demarcation between curative and non-curative stages. These nurses suggested that the characteristics of non-haematological cancers make it easier for clinicians to determine when to stop treatment and begin planning end of life care.

*‘I think with oncology patients … it's a gradual decline and they can kind of … decide when enough is enough … but haematology is really fast, in that they become unwell and … the time that they spend when it would be like their end of life period, you spend trying to save them, and then all of a sudden … they do die really quickly.’* (Haematology Nurse 8)

Some haematologists were perceived as generally reluctant to discontinue chemotherapy until all options had been explored, so they could be assured they had *‘tried everything’* to meet (particularly younger) patients' hopes and expectations for cure. Uncertain outcomes, and the increasing availability of new treatments, were said to be associated with prolonged and intensive therapy that could leave patients too depleted for home transfer after chemotherapy cessation.

‘*while there's the newer and novel agents, there is always that possibility, that you could try this, we could apply that*’ (Haematology Nurse 4)

‘*for most of them [patients], I think there is unrealistic expectations about outcome…and they [patients] persevere and persevere*’ (Haematology Nurse 3)

‘*they would be too unwell to go home, you wouldn't get them home*’ (Haematology Nurse 1)

Escalation of treatment (e.g. use of continuous positive airway pressure – CPAP; admission to high dependency/intensive care units) in advanced disease, was perceived by nurses as contentious, and linked to increased risk of hospital death. Despite showing awareness of the difficulties haematologists face making decisions about critical care interventions, one interviewee commented that ‘*just because you can do something, it is not necessarily the right thing to do’*. In the nurses' experience, patients often died in intensive care, and instances were recalled when haematology and intensive care doctors seemingly held differing perspectives regarding the risks and benefits of escalation.

*‘the registrar said, “I want to put him on some CPAP”. Now they had got a DNR [do not resuscitate order] in place with this patient … he was over 80 … and they [intensive care unit consultant] said he wasn't to go to ICU … the ICU people didn't think it was appropriate, so he didn't end up getting it’* (Haematology Nurse 2)

### 4.2 Close clinician-patient bonds

Nurses frequently referred to the strong bonds between patients and the clinicians looking after them, due to lengthy periods of in-hospital treatment and outpatient follow-up. Patients and family members were described as feeling *‘safe and secure’* in the care of staff whom they know and trust, and *‘at home’* in the hospital environment. The majority of interviewees regarded hospital as the preferred place of care/death for patients (and their relatives).

‘*the family have got so close to us, the patients have got so close to us, it feels quite normal [for them] to be in hospital…it just feels the right place to be*’ (Haematology Nurse 7)

‘*a lot of people choose to die in hospital…they just feel safe…this is their second home*’ (Haematology Nurse 8)

Nurse participants appeared to derive high levels of satisfaction from providing holistic care to patients *‘from diagnosis until the end of their journey’*, and described themselves as frequently unwilling to *‘let go’* of *‘their’* patients after treatment cessation. Reluctance to hand over to palliative or district nursing care sprang from concerns patients might feel they were *‘washing* [their] *hands’* of them after treatment failure, and that patients would lack time to form relationships with *‘new’* nurses following hospital discharge.

‘*we haven't been good at letting go of our patients … we like looking after them from the cradle to the grave … When it becomes palliative, in terms of going to a different ward, different environment … it's difficult … for the nurses …, relatives …, patient …, doctors …, relationships have been forged …, the end of life periods might be [too] short … to forge [new] relationships'* (Haematology Nurse 4)

*‘somebody new … people coming in at the very end, when they often feel, oh, why is that, are you done then with me, are you washing your hands of me?’* (Haematology Nurse 5)

### 4.3 Delayed end of life discussions

Haematologists' reluctance to *‘give up’* on patients, and patients' wishes to continue treatments that offer hope for remission or cure, were identified as contributing to delayed end of life discussions. Younger patients were often described as *‘in denial’* that death might be an outcome, and reluctant to engage in end of life conversations.

‘*the patients look to the doctors to kind of save them…When you have got a patient asking for help, it's difficult to say, I don't think you are going to survive this, you should go home*’ (Haematology Nurse 1)

‘*I was trying to find out her preferred place [of death] and she was kind of blocking it all…she was in denial about it*’ (Haematology Nurse 6)

Nurses emphasised the need for early, frank discussions between haematologists and patients/family members about likely treatment outcomes, to avoid raising unrealistic expectations.

*‘I think there is a lack of honesty about what the treatment will do for the patient … if you're given the option to have chemo you are going to do that, unless it's presented as “we could do this, but actually it won't be effective for you”’* (Haematology Nurse 3)

While recognising that identifying the appropriate time to begin talking to patients about end of life preferences was difficult for haematologists, nurses regarded timely elicitation of this information as essential to allowing sufficient time to organise end of life care at home or in hospice. Delayed discussions were said to result in hospitalised patients becoming too ill to consider alternative end of life options, and therefore remaining in hospital to die.

‘*I was thinking we should be having this [end of life] conversation with him, but he was too unwell*’ (Haematology Nurse 6)

‘*sometimes patients can deteriorate very quickly…often it's difficult to get them there [hospice] then, so I think it's about…trying to broach the subject, a bit sooner*’ (Haematology Nurse 7)

Delays initiating ‘difficult’ discussions with patients were partly attributed to varying levels of confidence amongst haematologists.

*‘I've witnessed some excruciating conversations and some fantastic ones, so it is, it's entirely dependent on who does it.’* (Haematology Nurse 5)

Nurses commented that sustained and close contact with patients and relatives often alerted them to small changes in a patient's condition, which doctors might be unaware of, and provided them with insights (e.g. into preferences) they regarded as useful during multidisciplinary team meetings. Most attended such meetings regularly, and felt their participation was welcome and valued, though a minority reported negative reactions from medical staff to suggestions about considering switching to a palliative focus to care.

‘*we discuss a lot of treatment options and outcomes…and people get their say…and I think the nurses on the ward are listened to…their comments are taken into consideration…* ’ (Haematology Nurse 5)

‘*to be honest, sometimes they [haematologists] are very dismissive of us…I mean I was made to feel I want to put everyone onto the LCP [Liverpool Care Pathway]…and I get belittled for that…* ’ (Haematology Nurse 2)

### 4.4 Lack of integration of palliative care services

Nurses reported wide variation in haematologists’ palliative care referral patterns, and drew comparisons with the management of patients with other cancers, where referrals usually occur much earlier in the pathway.

‘*one consultant may be very good at putting people on palliative care…and fast track discharging…and a different consultant doesn't agree and thinks he's trying to kill them by not giving more treatment’* (Haematology Nurse 3)

‘*there's a very definite split between haematology and oncology…oncology moves on…to palliative care services maybe earlier than haematology…With some of the haematology patients, that doesn't happen until the very last hours or very last days of life…* ’ (Haematology Nurse 4)

With the exception of severe pain in myeloma, patients were said to rarely experience the complex symptoms specified in criteria for referral to hospital specialist palliative care teams.

*‘palliative care didn't want to come and see him because they were saying he didn't have complex needs at that point’* (Haematology Nurse 3)

The nurses regarded themselves as capable of managing most end of life symptoms, and able to provide emotional support to patients and family members.

*‘we manage their anaemia and thrombocytopenia … most of it we can manage ourselves … If there's symptoms that we can't manage, cause the myeloma patients and the lymphoma patients can have a lot of sort of pain … they need more input really … The leukaemias don't tend to have too much of a problem with that … ’* (Haematology Nurse 5)

However, caring for dying patients on a busy acute haematology ward could be difficult due to time constraints. Moreover, nurses were concerned about losing contact with ‘outliers’, transferred to other hospital wards to facilitate admission of acutely ill patients.

‘*we're just so busy, sometimes only 2 people on at night for 20 patients and if you've got someone who's dying, it's crap, it's awful*’ (Haematology Nurse 1)

‘*sometimes we have to move patients off here [haematology ward] to get people in that need chemo…The doctors would go round and see them anyway every day…it's just the nurses they wouldn't have that relationship with…* ’ (Haematology Nurse 8)

Some participants had access to beds dedicated to the provision of palliative care in the hospital where they worked, which were close to the specialist ward/unit and so allowed haematologists to maintain contact with patients. This was seen to facilitate good end of life care, but was not available in all settings.

*‘it's a little 18 bedded unit, very specially for haematology and oncology … following treatment and for giving [supportive] treatment so yeah, it's definitely been a great improvement’* (Haematology Nurse 7).

Hospice services were said to be *‘rarely’* accessed. Reasons given for this were patients' lack of awareness or negative perceptions of hospice, coupled with disinclination to leave haematology care.

*‘making the decision to go somewhere that's quite new, at that time … that's hard … it's about convincing people that is the best place for them to go … If they're well enough, we ask them to maybe visit the hospice with the family … it's the unknown that's frightening’* (Haematology Nurse 7)

Lack of available hospice beds was also problematic, particularly when a bed was required at short notice and the patient rapidly deteriorating.

‘*there's a finite amount of beds available at the hospice’* (Haematology Nurse 4)

‘*sometimes patients deteriorate very quickly so by the time the bed becomes available they are not well enough to travel’* (Haematology Nurse 7)

Inconsistent policies concerning administration of supportive therapies (e.g. blood product transfusions), was said to further compounded difficulties in accessing hospice care.

*‘they [hospice] won't take them [patients with haematological conditions] if they need blood and platelets'* (Haematology Nurse 3)

### 4.5 Barriers to death at home

Perceived barriers to home death included delays in hospital discharge; variable availability of community nursing services; role of family members; loss of GP contact; and poor communication across care settings.

#### 4.5.1 Delays in hospital discharge

The need for early elicitation of preferences to facilitate organisation of community support for patients wishing to die at home was repeatedly emphasised. Short notice discharge could pose almost insurmountable problems for nurses, due to limited time for completion of paperwork and liaison with community services. Shared responsibility for discharge across the nursing team, and use of fast track processes were said to help; but even securing an ambulance to transport patients home, and being able to ‘spare’ an accompanying nurse, could be difficult.

‘*my heart almost sinks when the patient says‘I'd like to go home…it takes up an incredible amount of time…phone the hospice, phone the district nurses, get stuff in place, like a bed, sometimes you don't have time to do it…and the patient is deteriorating in front of your eyes…Sometimes, a lot of the time, you have to stop and say, you are too unwell to go home now. I think that's one of the reasons people don't die at home…if you get someone home, you see it as a major achievement’* (Haematology Nurse 1)

‘*I couldn't get an…ambulance…I thought he was going to die on the way…I couldn't go in the ambulance with him…* ’ (Haematology Nurse 3)

Nurses commented that patients whose discharge occurs at a very late stage often become too ill to leave hospital before arrangements are complete, or die soon after arriving home.

*‘most people, if they say, I want to die at home, they don't literally mean, I want to be whisked home hours before I die … I imagine people want to spend a bit of time at home and then die … not just literally get shoved through the door’* (Haematology Nurse 2)

#### 4.5.2 Variable availability of community nursing services

District and palliative care nursing was perceived as *‘patchy’*, with gaps in out-of-hours care being cited as a major reason for patients being unable to leave hospital, or readmitted. The Marie Curie night sitting service (a registered UK charity providing nursing care for people with life-limiting diseases) was viewed as an extremely valuable source of support, but was often said to be limited.

*‘community is very difficult in terms of them being staffed in a way that patients can go home and be fully supported … The difficulty obviously would be evenings and weekends … We have delayed discharges of people wanting to go home because we have not been able to get as many carers in as we would need’* (Haematology Nurse 4)

Relatives, often anxious due to lack of overnight support, were described as likely to contact out-of-hours’ services, or the haematology ward if the patient's condition deteriorated, resulting in patients discharged home for end of life care *‘bouncing’* back into hospital.

*‘People are in pain during the night, people are afraid during the night, people are awake during the night and a lot more of it is magnified so much more during the night than it usually is during the day, when they've got lots and lots of people around.’* (Haematology Nurse 4)

*‘when the patient has difficulty at home … people bounce back in and end up dying where they didn't particularly want to be’* (Haematology Nurse 4)

#### 4.5.3 Role of family members

Haematology nurses commented that patients were often more, and relatives less, likely to express a preference for home death, making planning end of life care difficult.

*‘it [dying at home] might be the patient's choice … the family might be dead against it*’ (Haematology Nurse 9)

*‘I think they [family members] feel scared at the thought of having them [patients] at home … they often say, we'd rather be in hospital’* (Haematology Nurse 7)

Having family able and willing to provide care was regarded as essential for home care/death, but was not always available (e.g. patients with frail/working relatives, living alone or not having relatives nearby).

*‘it's often people that don't have family support that can't be managed at home for end of life care … because they just need somebody. The services that we get, the maximum is four calls a day, so in between those calls, they're on their own … So unless they've got a family member who can … sort of, be around and in and out, then it's impossible for people to really safely be at home’* (Haematology Nurse 5)

*‘people are working … years ago they would rally round to take care of their loved one’* (Haematology Nurse 1)

Nurses shared a perception that many people have unrealistic expectations of death at home, based largely on media reports. They commented that onset of symptoms (e.g. dyspnoea and bleeding), could provoke significant distress in patients and relatives, and shift preferences from home to hospital-based care.

‘*in the media…the reality of dying at home is not portrayed…it's very different from the“home sweet home”image*’ (Haematology Nurse 4)

‘*a lot of haematology patients are quite worried about bleeding…and how that would be managed at home…I think that kind of frightens them…* ’ (Haematology Nurse 7)

Parents of young children in particular were thought to often prefer patient death to occur outside the home, in an attempt to mitigate distress.

*‘you've got the dying person in the living room … the patient and wife or husband are thinking, we can't do that, it's too scary for the children … Practically, it's a lot easier for them to be … in hospital … or go to the hospice’* (Haematology Nurse 1)

#### 4.5.4 Loss of contact with GP

Loss of contact between patients and GPs was attributed to the predominant role of haematologists in providing care. GPs were perceived as often *‘out of the loop’* and lacking current information about treatment and progress.

‘*they might not see the GP for two years…they would rather we deal with it all’* (Haematology Nurse 1)

‘*we don't discharge palliative patients [to GP]’…in terms of going home, they still remain in our system*’ (Haematology Nurse 4)

Several nurses noted that it is *‘drilled into’* patients to contact haematology specialists, rather than their GP, for any problems relating to their cancer.

‘*when they're first diagnosed…we kind of instil it into them that they must ring if they've got any problems and not to ring the GP…we are the ones doing the chemo and know about the side effects…it's drilled into them to ring us about everything…I can see how the GP does lose contact*’ (Haematology Nurse 7)

‘*they [patients/relatives] have had a lot of contact with hospital and then if they [patients] become unwell…they'll often contact us first, first line of support, and end up getting admitted’* (Haematology Nurse 6)

#### 4.5.5 Poor channels of communication across care settings

Poor channels of communication across the primary/secondary care interface, including incompatible electronic record systems that, one participant said, *‘don't speak to each other’* were considered to impede achievement of home death. ‘*Flagging’* patients in secondary care, via a system similar to that within the Gold Standards Framework (‘Gold Standard Framework Proactive Identification Guidance (PIG) Registration Form’) (www.goldstandardsframework.org.uk), was suggested as a potential means of alerting primary care staff to people possibly in the last year of life, who are likely to require GP, community nurse and out-of-hours support, if they want to die at home.

*‘primary and secondary care historically have never spoken to each other very well. Communication has been very poor … we need to tighten that up. We can't see what's gone on at home, the same way the GP can't see what we've discussed here [hospital] … Some sort of system where you could [access] community [and] hospital [records] … would be perfect’* (Haematology Nurse 4)

An absence of systematic and routine recording of end of life discussions, resulting in the need to re-initiate sensitive conversations that had already taken place, was also noted.

*‘I think documentation could be better … so that everybody knows what's been said and when, and clearly documented … what the preferred place of death was, or why a discussion hadn't taken place … then you could go straight to the notes … and everyone would be aware of this decision’* (Haematology Nurse 5)

## 5 Discussion

A series of complex, interrelated factors were identified by haematology nurses as contributing to hospital death among patients. The most significant relate to the disease characteristics (unpredictable trajectories, indistinct transitions, difficulties with prognostication), their often intensive treatments and uncertain outcomes. Other factors were also described, some of which were considered more amenable to modification (e.g. availability of community nursing services, night sitters and hospice beds). Earlier discussions, focussing on realistic outcomes, better integration between specialities and improved communication channels across settings were factors perceived as having the potential to improve end of life care and facilitate achievement of preferences.

A further reason for hospital death identified by nurses, was that patients may prefer to die in this setting, due to the close bonds between haematology staff, patients and family members. Relatives' preference for patient death in hospital was also linked by nurses to anxieties concerning the adequacy of home care and their own ability to provide care. This reflects findings from an interview study with family members providing home care (Totman et al., 2015), that revealed intense feelings of responsibility, isolation and anxiety. Furthermore, a narrative literature review (Morris et al., 2015) highlights caregivers’ own needs for emotional support, information and practical assistance with hands-on care.

Continuation of treatment until close to death was considered a major contributor to hospital death in our study, though often perceived as unavoidable, particularly in younger patients receiving intensive chemotherapy for aggressive disease. Administration of treatment close to death is considered overly aggressive by some (Beaussant et al., 2018; Hui et al., 2014), but may be due to haematologists’ concerns that the potential for cure still exists (even in advanced disease), the availability of multiple and/or new lines of treatment and difficulty identifying refractory disease and the end of life phase (Hui et al., 2015; McCaughan et al., 2017; Odejide et al., 2014); it may also be based on often unrealistic hope for response or cure, driven by patients and clinicians, as described in our own study and elsewhere (Laryionava et al., 2018; Odejide et al., 2016).

Nurse participants regarded decisions to escalate treatment as contentious and linked to increased likelihood of hospital death, particularly in elderly patients with advanced disease. Escalation has been described by others as complex and emotive (Hill, 2010), with tensions potentially arising between clinicians due to differing perceptions of risk and benefit, as noted by our interviewees. Difficulties arise because patients are generally severely ill on referral to critical care, and survival often poor (Azoulay et al., 2015; Cheng et al., 2016; Hill, 2010). Although these issues have resulted in calls for timely communication and close collaboration between teams (Cheng et al., 2016), a recent UK study suggests that the appropriateness of escalating care is often not considered until patients are acutely unwell and outreach teams intervene (Pattison et al., 2015). Current UK guidance recommends, however, that certain haematology patients (i.e. with good performance status) are considered for intensification (Wise et al., 2015).

Participants said their sustained contact with patients and relatives gives them unique insights into the values and preferences of individuals, as well as changes in a patient's condition that should be considered during decision making. However, two said their perspectives were neither routinely sought nor appeared valued by colleagues, causing them to feel angry and upset. Canadian haematology nurses have described how their in-depth patient knowledge facilitates early recognition of dying, but how negotiating the contradictory tasks of fighting disease while preparing patients for end of life can cause both personal and professional conflict, distress and burn-out (Leung et al., 2012). Similar existential, moral and ethical difficulties have been reported in studies investigating the role of critical care nurses in Canada and the USA (Bach et al., 2009; Robichaux and Clark, 2006), and further UK research into these issues is warranted.

The notion of dying at home as a quality indicator of a ‘good death’ is increasingly coming under scrutiny, and indeed may not be feasible, or the main priority in all circumstances, for all people (Gomes et al., 2015; Holdsworth, 2015; Pollock, 2015). Hospitalisation is regarded by some as potentially unavoidable late in the cancer trajectory, however, with optimisation of inpatient care a key priority at this time, more so than place of death (Numico et al., 2015). Little is known about preferences of patients with palliative care needs for place of care during periods of acute illness, or of any benefits they (and their family members) may experience from hospitalisation at end of life (Robinson et al., 2015). In our study, nurses commented that end of life experiences may differ for patients in hospitals lacking a dedicated haematology ward, or who are outliers in other areas. They commented that caring for dying patients alongside those who are acutely ill and receiving treatment can be challenging and alluded to time pressures in acute environments as constraining their capacity to meet the needs of dying patients and their relatives, particularly for psychological and emotional support in relation to existential distress. Currently, it is not known whether haematology nurses feel adequately prepared to provide end of life care, either educationally or emotionally (Bloomer et al., 2013) as research exploring such issues is lacking in the UK.

Frank, early and realistic discussions regarding treatment outcomes and possible cessation, were perceived as vital for successful advance planning, particularly where home death is preferred. Delayed discussions were primarily attributed by nurses to prognostic uncertainty and lack of clear indicators of disease progression, with resultant challenges for haematologists in identifying the best time to initiate such conversations. Our study interviewees also reported variation in haematologists' confidence in commencing ‘difficult’ discussions. A recent UK review reports varying levels of satisfaction among haemato-oncologists with their communication skills training and little evidence of consistency in teaching beyond medical school and specialist training (Christie and Glew, 2017). Such interventions are recommended, however, if the communication challenges faced by haematologists are to be met (Dharmarajan et al., 2016; Odejide et al., 2016; Christie and Glew, 2017).

A further difficulty is deciding if/when to refer patients to palliative care services (Auret et al., 2003; Manitta et al., 2010). Nurses in our study reported varying levels of collaboration between haematology and palliative specialists and inconsistent referral practices, although recent studies indicate increasing interdisciplinary working and co-location (McCaughan et al., 2018; Wright and Forbes, 2014). The nature and extent of the haematology nurses’ involvement in referral decisions was unclear, as was their role in providing palliative care, underscoring how little is known about the role of nurses in these activities (Kirby et al., 2014), despite the importance placed on team-based approaches to managing timely and effective transitions.

The benefits of early palliative care for patients with blood cancers (mitigating symptoms, providing psychosocial support and guiding patients’ expectations) are increasingly acknowledged (Boucher et al., 2018; Hochman et al., 2018). Some authors, however, report that under-utilisation of this service is likely to persist until palliative care is conceptualized as an “extra layer of support”, additional to standard care, and alongside active treatment (LeBlanc et al., 2017). The appropriateness and provision of palliative care should not be dependent upon a particular prognosis, which leads to late or absent referrals of patients to palliative care (Beaussant et al., 2018).

Our findings concur with other UK studies indicating that haematology patients did not always have complex symptoms requiring specialist management (with the exception of myeloma), that access to specialist palliative care services may be curtailed by strict referral criteria and that inpatient palliative care beds were lacking (McCaughan et al., 2018; Wright and Forbes, 2014). Such limited access (compared to other cancers) may unintentionally reinforce patient preferences to remain with the haematology team for end of life care. Moreover, early palliative care is reportedly feasible and effective for haematology patients (El-Jawahri et al., 2016; Porta-Sales et al., 2017) and could familiarise them with this facility, thus dispelling some of the negative perceptions said to contribute to under-use of services (Wright and Forbes, 2014; Zimmermann et al., 2016). Interviewee suggestions for increased access to hospital palliative care beds echo those of haematologists (Wright and Forbes, 2014).

Barriers to patients with haematological cancers accessing hospice care (inconsistent transfusion policies, lack of rapid-access beds), are widely reported in the UK (Brown and Bennett, 2007; Wright and Forbes, 2014), the US (Odejide et al., 2017), and elsewhere (Cheng et al., 2015), and were also identified in our study. Treatment viewed as ‘symptom control’ by haematologists is often considered ‘active’ by palliative care physicians, and not provided in hospices (Auret et al., 2003). Although evidence is lacking (e.g. regarding palliative transfusions), the need to develop guidelines to simultaneously meet curative/life-prolonging and palliative care needs has been emphasised (Chin-Yee et al., 2018; Epstein et al., 2012).

Many barriers to home care identified in our study (e.g. unavailability of carers, gaps in community nursing services) are applicable to other cancers (National End of Life Care Intelligence Network, 2015; Sarmento et al., 2017). Limited evidence exists about informal carer perceptions of their links with healthcare professionals providing end of life care in the home setting (Holdsworth, 2015; Seamark et al., 2014; Shepperd et al., 2016). A recent Australian study, however, illuminates the vital role family, friends and neighbours play in supporting home death and raises questions about optimal integration of informal and professional systems (Leonard et al., 2018). A UK study reports the important role of community nursing in the last three months of life in promoting achievement of home death (Gomes et al., 2015). However, while improved home palliative care may enhance patient and carer experiences (Sarmento et al., 2017), it is unlikely to prevent hospital admission of patients whose condition deteriorates suddenly and acutely (Numico et al., 2015). Delivery of optimal end of life care to patients with haematological malignancy, in line with their stated preferences for place of care and death, demands highly responsive, multidisciplinary teams to collaborate closely, across community, hospital and hospice settings (Pasquale et al., 2006, 2014; 2015).

Unrealistic expectations concerning dying were considered a barrier to home death by our study nurses. Patients and relatives were described as unprepared for the patient's deterioration, and fearful about how and when death might occur, undermining relatives' confidence to provide care. Numerous studies have similarly reported high levels of anxiety among home-carers regarding uncertainty about death (Harding et al., 2012; Martín et al., 2016; McIlfatrick et al., 2018). A systematic review highlights carers' desires for written information about disease progression, and training to enable them to feel more competent in their role (Ventura et al., 2014). These findings are supported in a large Canadian study with bereaved caregivers, who said they valued being taught what to expect as the patient's condition declined and how best to manage symptoms (Bainbridge et al., 2017).

Poor communication channels between haematology and primary care were linked by nurses to hospital readmission after patient home-discharge for end of life care. Use of shared platforms across settings could promote timely access to information, facilitate faster community palliative care services set up, and increase the likelihood of home death. Electronic Palliative Care Coordination Systems (EPaCCs) are currently in use in the UK to support recording up-to-date preferences of patients thought to be in the last year of life. This system is aimed at GPs, out-of-hours/emergency services, nursing/palliative care teams, hospitals, hospices and care homes; views about utility are mixed however (Petrova et al., 2018; Wye et al., 2016).

It is important to clarify prognostic expectations and patient preferences despite the difficulties we describe. Routine use of the Gold Standards Framework Proactive Identification Guidance (GSF PIG) (www.goldstandardsframework.org.uk) and Supportive Palliative Care Indicators Tool (SPICT) (www.spict.org.uk) could help identify patients in their last 12 months of life, and facilitate achievement of preferences. O'Callaghan et al. (2014) found high sensitivity, specificity and predictive value of the GSF PIG in screening for end of life in their cross sectional study of 501 hospitalised patients with heart and renal failure, and a study designed to evaluate SPICT has similarly shown promising results, though further research is needed (Highet et al., 2014). Haematology specific tools are currently lacking (Moreno-Alonso et al., 2018), but would undoubtedly be useful, particularly as so many patients with these diseases die well within a year of diagnosis. Another initiative being rolled out in the UK is the Recommended Summary Plan for Emergency Care and Treatment (ReSPECT) (www.respectprocess.org.uk) which aims to improve information sharing across settings and enable patient preferences to be met in emergency situations, when rapid decision-making is required; the impact of this remains to be seen.

### 5.1 Strengths and limitations

To our knowledge, this is the first UK study to investigate haematology nurses' views of their patients' places of care and death. In-depth interviews yielded ‘rich’ data, and inclusion of nurses from different settings facilitated capture of a range of perspectives. The number of nurses included was small, but not atypical of qualitative descriptive studies, which rely on the quality of data collected for validity, rather than the number of included participants (Kim et al., 2017). The aim of our study was relatively narrow; we recruited a specific sample of nurses with the expertise to inform the research question; the interview dialogue was of high quality; and the analytical strategy incorporated in-depth exploration and interpretation of data to generate new insights and understanding. Whilst we recognise that the small sample size requires caution in extrapolating findings, the relatively under-researched nature of the topic means this is less important than ‘sensitizing’ readers to new information (Green and Thorogood, 2018). Further studies are required with larger samples of nurses working in diverse service configurations, and in different localities.

## 6 Conclusions and implications

Haematology nurses attributed hospital deaths to several inter-related factors; the most significant being the intensive nature of treatments, and the propensity for sudden deterioration and death. Prognostication was acknowledged as challenging, and hospital death was often perceived as unavoidable, and the preferred option for some. Earlier, frank conversations about end of life care, focussing on realistic outcomes; closer integration of palliative care and haematology services; better channels of communication across settings, adequate community nursing services and hospice beds, and hospice policies aligning life-prolonging and palliative care, were perceived as having the potential to improve end of life care and the likelihood of home or hospice death, where preferred.

## Conflicts of interest

The authors declare they have no conflict of interest.

## Availability of data and materials

All data and materials relating to this research are from the Haematological Malignancy Research Network. Data are not publicly available due to the risk of participant identification from specific contexts revealed when reading entire transcripts and due to the terms and conditions regarding the release of data to third parties upon which ethical approvals for this study were contingent. Reasonable requests for further information relating to this data can be made to the corresponding author.

## Funding

This work was supported by the Marie Curie Research Grants Scheme (Grant Reference: C38860/A12554). HMRN is funded by Bloodwise (Grant Reference: 10042).

## Disclaimer

This paper presents independent research supported by Marie Curie Cancer Care. The views expressed are those of the authors and not necessarily those of the funder.
